# Supporting New Graduate Nurses’ Information Seeking: Perspectives of Nurse Managers and Senior Nurses in Japanese Hospitals

**DOI:** 10.3390/nursrep16050153

**Published:** 2026-04-30

**Authors:** Misuzu Gregg, Chifuyu Hayashi, Masami Tamada

**Affiliations:** 1 Graduate School of Nursing, Meio University, Nago 905-8585, Japan; 2 Graduate School of Nursing, Hyogo Medical University, Kobe 650-8530, Japan; ch-hayashi@hyo-med.ac.jp; 3 Department of Nursing, Kobe City College of Nursing, Kobe 651-2103, Japan; masami-tamada@kobe-ccn.ac.jp

**Keywords:** new graduate nurses, information seeking, nurse managers, senior nurses, support

## Abstract

**Background/Objectives:** Effective information seeking is essential for new graduate nurses’ adaptation to the workplace. The objective of this study was to identify how nurse managers and senior nurses support new graduate nurses’ information seeking and the expectations underlying such support. **Methods:** Nurse managers and senior nurses from Japanese hospitals participated in semi-structured interviews. Data were analyzed using qualitative content analysis by coding meaningful units, grouping similar codes into subcategories, and organizing them into broader categories. Ethical approval was obtained from the research ethics committee of the first author’s institution, and written informed consent was obtained from all participants. **Results:** Participants included twelve nurse managers from five hospitals and fourteen senior nurses from three hospitals. The nurse managers had an average of 7.4 years of supervisory experience, and the senior nurses had an average of 14.2 years of clinical experience. Participants expected new graduate nurses to demonstrate appropriate attitudes toward seeking information, engage in proactive communication to express their needs or uncertainties, take initiative in seeking and obtaining information, and build relationships with senior nurses that would facilitate information seeking. To support these expectations, participants adopted approaches that encouraged information seeking, proactively provided necessary information, and promoted information sharing among peers. Nurse managers also sought to create a work environment in which all staff members collectively nurtured new graduate nurses. **Conclusions:** To facilitate information seeking among new graduate nurses, nurse managers and senior nurses need to foster a supportive work environment. They also need to recognize the information content, sources, tactics, and timing appropriate for new graduate nurses.

## 1. Introduction

In Japan, registered nurse qualification requires completion of basic nursing education offered mainly through four-year university programs or three-year programs at junior colleges and vocational schools. They must then pass the National Nursing Examination and obtain a nursing license, which does not require renewal. Since 2010, hospitals have been encouraged to provide clinical training programs for new graduate nurses to promote the acquisition of essential practical competencies for clinical practice. By 2020, nearly all hospitals in Japan had implemented these training programs in accordance with the government’s “Guidelines on Clinical Training for Newly Graduated Nursing Personnel” [1].

The smooth transition from nursing student to new graduate nurse is an important issue in Japan, as it is in other countries. According to the Japanese Nursing Association, 7.6% of new graduate nurses left their jobs within one year in 2017 [2]; in recent years, this figure has been reported at around 10% [3,4]. These rates are lower than those reported in some other countries; for example, South Korea reported a turnover rate of 44.5% in 2020 [5], and the United States reported 31.7% in 2022 [6]. Although the turnover rate in Japan is comparatively low, leaving the first hospital within one year can still have a significant impact on new graduate nurses’ careers.

The difficulties faced by new graduate nurses during the transition period are described as transition shock [7]. This refers to the acute phase of professional role transition experienced by new graduate nurses as they adjust from the protected environment of academia to the complex, unpredictable, and demanding context of professional nursing practice. It is characterized by emotional distress, physical exhaustion, intellectual overload, and sociocultural adjustment. Effectively coping with transition shock is critical for new graduate nurses as they progress toward full professional functioning. Transition is often regarded as the initial phase in which newcomers begin to integrate into their new professional and organizational contexts, thereby initiating the process of organizational socialization [8]. Organizational socialization is defined as the process by which an individual acquires the social knowledge and skills necessary to assume an organizational role [9]. There has been a shift in organizational socialization research from an emphasis on organizational tactics, such as orientations and preceptorships, to a focus on newcomers’ proactive behaviors, which are self-initiated actions to learn about the new environment [10,11,12]. Although organizational tactics play an important role in newcomers’ socialization, proactive behaviors on the part of newcomers remain essential for their successful integration into the new environment, even when sufficient organizational support is in place.

Many empirical studies on proactive behavior have been conducted in non-nursing populations [10,13,14,15,16,17,18]; however, new graduate nurses also need to demonstrate proactive behaviors when they enter new work environments. Previous research on proactive behaviors among newcomers has identified information seeking, feedback inquiry, and relationship building as key forms [19]. Among these, information seeking has been conceptualized as a central and foundational proactive behavior during organizational entry [10,11], and is essential for organizational socialization [20,21]. Based on these studies, the present study focused on the information seeking of new graduate nurses. Information seeking is defined as “the intentional search for information necessary to achieve a particular purpose” [22] (p. 49). It reduces environmental uncertainty, facilitates understanding of what is expected by others, and is said to play an important role in workplace adaptation [23]. Building on our previous qualitative study that examined senior nurses’ expectations and support regarding new graduate nurses’ adjustment [24], the present study focuses specifically on information seeking as a distinct aspect of new graduate nurses’ work-related behaviors, with important implications for organizational socialization.

Accordingly, the objective of this study was to identify how nurse managers and senior nurses support new graduate nurses’ information seeking and the expectations underlying such support. The research questions of this study were as follows: (1) What expectations do nurse managers and senior nurses hold regarding newly graduated nurses’ information seeking? (2) What strategies do nurse managers and senior nurses use to support newly graduated nurses’ information seeking?

This study contributes to facilitating the organizational socialization of new graduate nurses and may ultimately support the delivery of high-quality patient care and their career development.

## 2. Materials and Methods

### 2.1. Study Design

This study employed a qualitative inductive design using qualitative description as the method, which aims to provide a comprehensive summary of events and experiences in the everyday language of participants. Qualitative description remains close to the data and offers a low-inference interpretation, making it particularly suitable when clear and straightforward descriptions of phenomena are desired [25]. Because this study aimed to describe nurse managers’ and senior nurses’ support for new graduate nurses’ information seeking in daily practice, as well as the underlying expectations associated with such support, a qualitative inductive design using qualitative description was considered the most appropriate methodological approach.

### 2.2. Sampling and Study Participants

We employed purposive sampling in this study. The inclusion criteria were nurse managers who supervised at least one new graduate nurse in their ward, and senior nurses with more than three years of clinical experience who were directly involved in teaching new graduate nurses. Participants were required to understand the purpose of the study and participate voluntarily. The exclusion criteria were nurse managers and senior nurses who were not involved in teaching new graduate nurses at the time of recruitment.

We sent research request letters to the directors of nursing at each hospital, explaining the details of the study, including its purpose, participant eligibility criteria, and ethical considerations. The directors of nursing then recommended potential study participants by sending their names to the first author via email. We subsequently sent invitation letters to these potential participants to request their participation. In total, 14 letters were sent to nurse managers and 20 to senior nurses. Twelve nurse managers and fourteen senior nurses agreed to participate in this study. To ensure voluntariness, we did not collect information on the reasons for non-participation. In the invitation letter, we stated that we are professors specializing in nursing education and nursing administration, described the purpose and rationale of the study, outlined the ethical considerations, and identified the organization that provided research funding. Those who agreed to participate returned signed consent forms prior to the interviews, as the interviews were conducted online. Immediately before each interview, the first author explained the study and ethical considerations again via the online platform and confirmed their consent.

### 2.3. Data Generation and Data Analysis

Data were generated through semi-structured interviews conducted by the first author, a female researcher with a PhD in nursing. Each participant was interviewed once using online videoconferencing platforms, including Zoom and LINE. Participants were asked to prepare a quiet, private room to ensure confidentiality. Some interviews were conducted at participants’ homes, while others took place in conference rooms or private offices in their hospitals. The first author met most participants for the first time, except for two senior nurses and three nurse managers. Two of the senior nurses worked at a hospital where the first author serves as an educational advisor, and the three nurse managers had graduated from the researchers’ former graduate school between four and fifteen years earlier. Although the first author had met these five participants before, potential influence was minimized because she had no personal relationship with them and was not in a position to evaluate their performance. Participants were also assured that none of the interview content would be shared with anyone at their hospitals.

The first author used an interview guide developed based on a literature review and the research team’s prior experience conducting interviews with senior nurses regarding workplace adaptation. We sought feedback on the interview guide from a nurse manager who had been responsible for nursing education across all nursing staff in a hospital for approximately 20 years. She reviewed and agreed with the interview guide. During the first interview with senior nurses, the first author also invited feedback on the interview questions. The study participant pointed out the need to clarify how key concepts, such as information and adjustment to the workplace, were defined for the study participants. In response, we added the following explanation to the interview guide: In this study, information is defined broadly to include diverse types of content relevant to practice. “Adjusting to the workplace” is understood as functioning as a member of the ward team. The interview guide was consistent across all interviews; however, as the interviews were semi-structured, the first author asked additional questions based on participants’ responses.

The interview guide was as follows:

1. New graduate nurses need to obtain various types of information to adjust to the workplace. What kinds of information do you think are necessary for them to do so?
2. From the perspective of new graduate nurses’ information seeking, how do you perceive their behaviors (e.g., aspects that are sufficient or insufficient)?
3. What kind of support do you currently provide to help new graduate nurses adjust to the workplace, particularly in terms of seeking information independently or receiving information? How effective do you think this support is?
4. What kind of additional support or improvements do you think are needed to better promote new graduate nurses’ information seeking?
5. What efforts or behaviors do you think new graduate nurses should demonstrate in seeking information?

During the interviews, the first author took field notes. With participants’ permission, all interviews were audio-recorded using a digital voice recorder and transcribed verbatim.

The data were analyzed using qualitative inductive content analysis. Data analysis was conducted manually using Excel and Word, without the use of specialized qualitative data analysis software. Each incident was coded, and the codes were organized based on shared meanings. Similar codes were grouped into subcategories, which were then further clustered and labeled to form overarching categories. The first author coded and categorized all data. Two co-researchers independently reviewed the coding and categorization. Discrepancies were discussed, and revisions were made until consensus was reached.

The study received ethical approval from the Research Ethics Committee of the first author’s affiliated institution (2023-009-1), and written informed consent was obtained from all participants prior to the interviews.

Confirming the rigor of this analysis, we used Lincoln and Guba’s criteria [26]. We asked three study participants, one nurse manager and two senior nurses, to review the analysis to establish credibility. All participants agreed with the analysis and indicated that it reflected their own experiences as well as those of other nurses in the same role. Reflexivity should be acknowledged, as the researchers’ backgrounds in nursing education and management may have influenced data interpretation. Efforts were made to enhance credibility through discussions among the research team. However, the research team has conducted related studies over many years and may share similar perspectives and underlying assumptions. This could introduce potential bias. To address this, the first author engaged in ongoing discussions with other researchers to critically reflect on her own assumptions and potential biases. These discussions were conducted without disclosing the actual interview data. We described the results in detail by presenting participants’ quotations to help readers understand the findings and to illustrate the context for transferability. To ensure dependability and confirmability, the entire decision-making process throughout the study was recorded and discussed among the authors, who have conducted qualitative research together for more than 15 years. The first author also has more than 25 years of experience teaching qualitative research to graduate students. In addition, an audit trail was maintained, including records of research procedures, the researcher’s intentions and perspectives, and the rationale for analytic decisions, enabling other researchers to trace the reasoning process. First, data from nurse managers and senior nurses were analyzed separately. However, because the expectations for new graduate nurses’ information seeking were similar across both groups, we combined the two datasets for the analysis of expectations.

Regarding data saturation, the first author judged that no new information was emerging by the 10th interview for both nurse managers and senior nurses, as interviews and data analysis were conducted concurrently. As twelve nurse managers and fourteen senior nurses agreed to participate, interviews were continued to further enrich the data. Since saturation is a matter of degree and there is always the potential for new insights to emerge [27], it is important to continue interviews to ensure adequate saturation. Data saturation was further confirmed after the completion of all interviews and data analysis. According to the literature [28], data saturation can be understood in terms of code saturation. Code saturation refers to the point at which no additional categories are identified in the data. The first author assessed code saturation following established approaches [28]. Using a subset of data from senior nurses regarding their expectations, a matrix was constructed to map categories across interviews. All categories had emerged by the 10th interview, and no new categories were identified in subsequent interviews, indicating that code saturation had been achieved. Based on this examination, further analysis of the entire dataset was not considered necessary.

## 3. Results

### 3.1. Characteristics of Study Participants and Hospitals

The twelve nurse managers were all female and had a mean of 7.4 years of supervisory experience across five hospitals. Eleven senior nurses were female and three senior nurses were male with an average of 14.2 years of clinical experience from three hospitals. The characteristics of the study participants are presented in Table 1 and Table 2. Three hospitals were acute care hospitals with an average of approximately 510 beds, and two were acute care hospitals that also had post-acute and community care wards, including convalescent rehabilitation and community-based integrated care wards, with an average of approximately 260 beds. The interviews lasted an average of 49.2 min (SD = 7.0; range = 40.6–77.1 min).

All five hospitals implemented a cascade mentoring system for nurses, in which senior nurses mentor junior nurses, who in turn mentor new graduate nurses. This layered framework, known as the Yanegawara method, is often metaphorically compared to overlapping roof tiles in Japan and facilitates the continuous transmission of clinical knowledge, technical skills, and professional values across successive generations of nurses. In this educational system, some senior nurses are referred to as ward-based nurse educators, while junior nurses serve as preceptors. Usually, each ward has one ward-based nurse educator. These nurse educators do not hold formal credentials or receive formal educational training. They are assigned this role in addition to their routine clinical duties and typically receive training within their hospital. In this study, the participants were charge nurses, ward-based nurse educators, and preceptors, all of whom are referred to as senior nurses.

### 3.2. Expectations for New Graduate Nurses’ Information Seeking

Nurse managers and senior nurses’ expectations for new graduate nurses’ information seeking indicated four categories and 13 subcategories. The four categories were demonstrating desirable attitudes toward seeking information, engaging in proactive communication to express one’s needs or uncertainties, taking initiative to seek and obtain information, and building relationships with senior nurses to enable information seeking (Table 3).

#### 3.2.1. Demonstrating Desirable Attitudes Toward Seeking Information

The study participants expected new graduate nurses to demonstrate a proactive and positive stance toward seeking necessary information, which they viewed favorably. This category consisted of three subcategories: showing willingness to learn, receiving guidance with a respectful attitude, and demonstrating diligence as a new graduate nurse.

*When I’m teaching, I can clearly see when a new graduate nurse is really motivated or has a strong attitude toward learning—for example, when they take notes or ask questions. I don’t think questions come up unless someone is genuinely interested, so when they do, I feel like, “Oh, this new graduate nurse is really eager to learn.” …I tend to think, “This is a new graduate nurse who is really worth teaching and able to actively seek information.*”

(Senior nurse No. 13)

She was a charge nurse with 14 years of nursing experience. Through years of experience teaching new graduate nurses, she came to recognize that a willingness to learn, a desirable attitude, was essential for seeking the information they needed.

#### 3.2.2. Engaging in Proactive Communication to Express One’s Needs or Uncertainties

The study participants expected new graduate nurses to proactively communicate their needs or uncertainties to receive support and to seek information to clarify those uncertainties. This category consisted of four subcategories: expressing one’s uncertainties or difficulties verbally, asking questions proactively when unsure, voicing one’s own thoughts even when unsure, and communicating needed information to senior nurses.

*When they ask about anything they don’t understand, we can also see what they don’t know. So, in that sense, it’s easier for the new graduate nurses to get the information they need, and it also makes it easier for us to support them. So it’s important to tell us what they don’t understand or what they need. After I understand what they need, I sometimes tell them to check it themselves, but I always explain what resources they can use*.

(Nurse manager No. 4)

The nurse manager expressed a clear expectation that new graduate nurses should actively seek necessary information through proactive communication rather than remaining passive recipients of instruction. She felt that when new graduate nurses express their needs or uncertainties, they can receive support and ultimately get the information they need.

#### 3.2.3. Taking Initiative to Seek and Obtain Information

The study participants expected new graduate nurses to make deliberate and proactive efforts to seek and obtain information independently. This category consisted of four subcategories: actively sharing information with peers, looking up unknown information independently, obtaining necessary information while collecting it with senior nurses, and identifying information-gathering methods suited to oneself.

*If they do not know what they are struggling with or why things are taking time, it is difficult to know how to support them. At the very least, I want them to first write down their concerns and questions and check things themselves. Then, if they say, “I’ve looked into this much, but I still don’t understand this part,” it becomes much easier to provide appropriate support and information*.

(Senior nurse No.6)

The senior nurse emphasized the importance of new graduate nurses taking initiative in seeking information. She noted that when new graduate nurses proactively identify uncertainties and seek answers independently, it helps senior nurses support them and enables new graduate nurses to acquire the information necessary for daily practice.

#### 3.2.4. Building Relationships with Senior Nurses to Enable Information Seeking

The study participants expected new graduate nurses to make efforts to build relationships with senior nurses in order to seek information. This category consisted of two subcategories: making attempts to initiate conversations with senior nurses and sharing personal information during casual conversations.

*It’s really just casual chatting with senior nurses who are a year or so ahead, but by joining in those conversations, I think you can start asking for information. Having chances to start talking with senior nurses who are close in age makes it easier, so building good relationships with senior nurses is really important*.

(Nurse manager No. 11)

The nurse manager emphasized that initiating casual conversations with senior nurses who are close in age provides key opportunities for new graduate nurses to obtain work-related information. Other study participants noted that new graduate nurses are expected to build good relationships with senior nurses, regardless of the senior nurses’ years of experience. Through getting to know one another and engaging in everyday interactions, new graduate nurses can foster trust and relationships that support information seeking.

### 3.3. Nurse Managers’ Direct Support for New Graduate Nurses’ Information Seeking

Nurse managers provided direct support for new graduate nurses’ information seeking, which consisted of five categories and eight subcategories. The five categories were as follows: encouraging information sharing among peers, supporting new graduate nurses to express their own thoughts, providing information about colleagues, teaching information-seeking methods, and providing information tailored to new graduate nurses’ current practice level (Table 4).

#### 3.3.1. Encouraging Information Sharing Among Peers

The nurse managers emphasized that information sharing among new graduate nurses is essential for obtaining the information they need. This category comprised a single subcategory that was identical to the category.

*I tell new graduate nurses, “Please make sure everyone receives this information.” For example, if there is a mandatory training session, I say, “Everyone needs to attend, so make sure you all read this. I am telling you now, so take responsibility and share it with everyone.” And they do pass it on to all their peers. Instead of simply saying, “Help each other and share information,” I try to specify exactly what information they need to communicate*.

(Nurse manager No.12)

The nurse manager observed that new graduate nurses tended not to share information effectively. Therefore, she told them exactly what information needed to be shared and hoped that they would eventually be able to share information effectively on their own.

#### 3.3.2. Supporting New Graduate Nurses to Express Their Own Thoughts

Nurse managers emphasized the importance of new graduate nurses articulating their thoughts in order to access necessary information. This category comprised a single subcategory that was identical to the category.

*When I sense that they seem to be struggling with something and I ask, “So, what’s going on?, “they start explaining their current situation in detail and with great effort. But when I ask, “What are you having trouble with?,” they just say, “Hmm…,” and cannot clearly identify it. Many of them don’t really know what they are struggling with, and that’s what troubles them. In most cases, it’s not about one specific issue; rather, they tend to have more general concerns. So, it’s important for them to talk about what they are thinking*.

(Nurse Manager No. 1)

The nurse manager had nineteen years of experience in the role and had learned to identify the times when new graduate nurses needed active support to voice their thoughts. She supported new graduate nurses in expressing their thoughts by modifying the way she asked questions.

#### 3.3.3. Providing Information About Colleagues

The nurse managers facilitated information seeking by providing information about the colleagues with whom the new graduate nurses worked. This category consisted of two subcategories: explaining senior nurses’ roles and responsibilities and providing information about physicians’ characteristics.

*I usually provide information about how many years of experience senior nurses have and what kinds of responsibilities they take on. During orientation, I also explain each senior nurse’s role on the ward—not their personality or character, but their professional role. For example, I might say, “She serves as a ward leader and is knowledgeable about heart failure, so you can consult her if you have related questions.” I try to share that kind of information with new graduate nurses*.

(Nurse manager No. 8)

She supported new graduate nurses’ information seeking by intentionally sharing information about senior nurses. Rather than leaving new graduate nurses to identify potential sources of information on their own, the nurse manager provided structured information about colleagues’ years of experience, professional roles, and areas of expertise. By clarifying “who knows what” within the ward, she helped new nurses identify appropriate individuals to approach for information.

#### 3.3.4. Teaching Information-Seeking Methods

The nurse managers themselves sometimes teach new graduate nurses how to seek information. This category consisted of three subcategories: teaching who to consult when unsure, teaching how to search for information in the electronic medical records, and teaching which reference materials to consult.

*While caring for patients, I showed them how to look up relevant information. For example, we would go to the computer together and search for information. I would say something like, “If the patient has a tracheostomy, you should check what kinds of cannulas exist. This patient is using this type, so find out what its structure is and how it works.” That was the kind of guidance I gave them*.

(Nurse manager No. 9)

The nurse manager had 16 years of experience in this role. She explained to new graduate nurses what kinds of information they needed to care for patients and how to find that information. Although the nurse manager’s primary responsibility was ward management, many of the study participants reported that they taught new graduate nurses how to find information whenever opportunities arose.

#### 3.3.5. Providing Information Tailored to New Graduate Nurses’ Current Practice Level

When nurse managers directly taught new graduate nurses what they needed for daily practice, they took into account the nurses’ current level of competence. This category comprised a single subcategory that was identical to the category.

*For things that new graduate nurses have not yet experienced, everyone needs to follow up and support them. I try to be mindful of this as well. For example, when caring for a patient who has become over-sedated, I explain how they should observe the patient and why those observations are necessary. I also ask questions and engage in back-and-forth exchanges with them as part of the teaching process, helping new graduate nurses understand the information they need for their daily practice in a way that fits their level*.

(Nurse manager No. 6)

The nurse manager adjusted the information she provided according to each new graduate nurse’s level of practice, offering step-by-step explanations and interactive guidance to help them understand essential information for daily practice.

### 3.4. Nurse Managers’ Indirect Support for New Graduate Nurses’ Information Seeking

Nurse managers also provided indirect support for new graduate nurses’ information seeking. Four categories and ten subcategories were identified. The four categories were as follows: creating a work environment where everyone nurtures new graduate nurses, mediating relationships between senior nurses and new graduate nurses, coordinating work assignments to allow interaction with various senior nurses, and supporting senior nurses in their educational role.

Nurse managers indirectly facilitated new graduate nurses’ information seeking by fostering an atmosphere in which all staff members supported and learned alongside them. They also managed assignments to promote interactions between new graduate nurses and senior nurses in the unit and when misunderstandings arose between senior and new graduate nurses, they clarified the intentions of both sides. In addition, nurse managers supported senior nurses’ education of new graduate nurses by advising them to share their reasoning processes and how to teach information-gathering skills.

### 3.5. Senior Nurses’ Support for New Graduate Nurses’ Information Seeking

The senior nurses’ support for new graduate nurses’ information seeking consisted of four categories and 10 subcategories. The four categories were adopting attitudes that facilitate information seeking, providing necessary information proactively, adapting teaching strategies to promote information seeking, and encouraging information sharing among peers (Table 5).

#### 3.5.1. Adopting Attitudes That Facilitate Information Seeking

The study participants adopted attitudes that made it easier for new graduate nurses to seek information. This category consisted of two subcategories: creating a comfortable atmosphere for information seeking and initiating conversations to make oneself approachable for consultation.

*I think creating an approachable atmosphere is really important. Since this is an acute care setting, when things get busy, we sometimes become tense or even say, “Can it wait?” But especially for new graduate nurses, I feel it’s important to interact with them in a way that avoids such responses. It’s about not coming across as intimidating, including the way we speak. I think it’s also important not to be dismissive or negative*.

(Senior nurse No. 5)

She worked at a comprehensive surgical center as a preceptor, where she emphasized maintaining an approachable attitude that enabled new graduate nurses to seek information in the high-pressure acute care environment. As similar perspectives were shared by nurses working in other wards, such as community-based integrated care wards and convalescent rehabilitation wards, senior nurses emphasized the importance of behaving in ways that encourage new graduate nurses to ask questions and seek information.

#### 3.5.2. Providing Necessary Information Proactively

The senior nurses played an active role in ensuring that new graduate nurses obtained the information they needed by actively sharing information. This category consisted of four subcategories: taking the initiative to share information, explaining what new graduate nurses need to learn, searching for information together, and teaching where information sources are located.

*I feel that new graduate nurses’ interactions with many different families increase substantially, and they often struggle with how to communicate in those situations. They frequently do not know how to explain things or what they should explain. At first, I usually have them watch me provide explanations. Before they go and speak with the family, I ask them to practice by saying aloud what they plan to explain, such as ‘I will explain this, this, and this.’ At this point, I add information if they need it. After that, we go together and they provide the explanation while I stay by their side. I offer additional explanation when it is needed*.

(Senior nurse No. 7)

She was a preceptor with 10 years of nursing experience. She provided the necessary information that new graduate nurses needed. Senior nurses, regardless of their positions or years of experience, also tried to provide information proactively when new graduate nurses needed it.

#### 3.5.3. Adapting Teaching Strategies to Promote Information Seeking

The senior nurses made creative efforts in teaching to promote new graduate nurses’ information seeking. This category consisted of three subcategories: considering the characteristics of new graduate nurses when providing guidance, verbalizing one’s own thinking while providing guidance, and checking understanding by asking questions about what new graduate nurses have learned.

*While observing alongside new graduate nurses, I make a point of saying things like, “This is the part you should be paying attention to.” I verbalize what I would normally watch silently, such as, “Right now I’m looking at this,” “This is the point I’m focusing on,” or, “This is the type of patient I would approach.” In other words, I try to share my thought process out loud as part of my teaching. I do this so that new graduate nurses can connect the information with their own understanding and, hopefully, transform it into meaningful learning*.

(Senior nurse No.11)

The senior nurse with six years of experience identified how verbalizing clinical reasoning helps new graduate nurses learn what information to look for and why. By making tacit judgment processes explicit, the senior nurse guides their information seeking in real time and supports them in developing meaningful, self-directed learning.

#### 3.5.4. Encouraging Information Sharing Among Peers

Similar to nurse managers, senior nurses encouraged information sharing among new graduate nurses to facilitate information seeking. This category had one subcategory, which is identical to the category itself.

*When one person shares their experience with three others, that information gets passed on so all three of them have it. Even if you haven’t actually experienced it yourself, you can still think, “Oh, so that kind of thing can happen.” I’ve even said before, “That’s something you should share.” It really creates those kinds of opportunities, so I feel sharing information among peers is really important*.

(Senior nurse No. 12)

Many senior nurses emphasized the importance of information sharing and therefore encouraged new graduate nurses to share information with their peers. Because new graduate nurses have limited experience, sharing information about nursing practice is a useful way for them to reflect on and consider future patient care.

### 3.6. Expectations and Support

**Figure 1:**
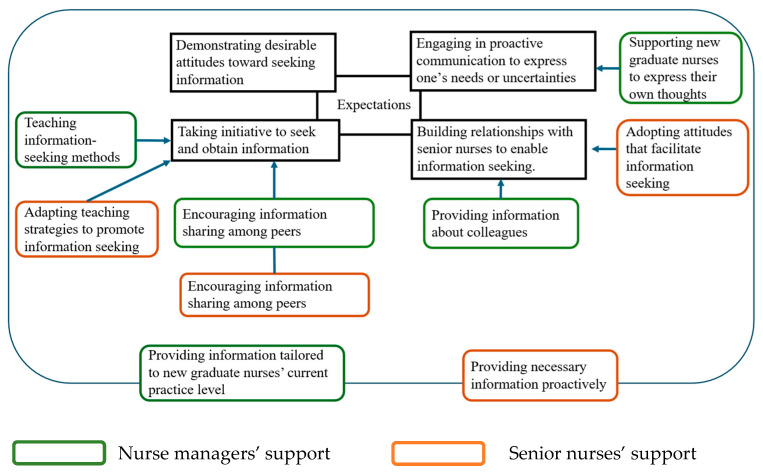
Expectations and Support.

Compared with the expectations identified, three of the four were reflected in the support: engaging in proactive communication to express one’s needs or uncertainties, building relationships with senior nurses to enable information seeking, and taking initiative to seek and obtain information. In contrast, the expectation that new graduate nurses demonstrate desirable attitudes toward seeking information, including respectful attitudes toward senior nurses and diligence as new graduate nurses, was not directly reflected in the support described. In particular, no explicit support specifically targeting these attitudes was identified in the data. Despite this, nurse managers provided information tailored to new graduate nurses’ current level of practice, and senior nurses proactively provided necessary information, regardless of these expectations (Figure 1).

## 4. Discussion

This study identified nurse managers’ and senior nurses’ support for new graduate nurses’ information seeking, as well as the underlying expectations associated with such support, which is an important factor in workplace adaptation. The study participants expected new graduate nurses to communicate their needs or uncertainties in daily practice in order to obtain the necessary information. Such articulation enables nurse managers and senior nurses to provide more appropriate support. Information seeking has been conceptualized as comprising four stages: problem identification, problem definition, problem resolution, and solution statement [29]. However, new graduate nurses do not always reach the stage of problem identification [30]. Sometimes, they do not fully understand patients’ conditions and are unsure whether they should consult senior nurses. This is because the knowledge they acquired in nursing school is not sufficient to apply to clinical practice. Therefore, the study participants understood this limitation and expected new graduate nurses to explain their circumstances, even when they were unable to clearly articulate specific problems. To actualize this process, it is important to create a work environment that facilitates information exchange and sharing among new graduate nurses, senior nurses, and nurse managers [31]. Senior nurses can facilitate information seeking by creating a comfortable atmosphere and initiating conversations that make them approachable for consultation. In addition, nurse managers’ indirect support in creating a supportive ward environment is also a valuable strategy.

This discussion distinguishes between what information is sought (information content) and where information is obtained (information sources) based on two references. Morrison categorizes information content into technical, referent, and normative information [18], while Lloyd and Somerville conceptualize information sources as textual, social, and physical modalities [8]. This distinction allows us to examine how new graduate nurses’ information seeking is supported by nurse managers and senior nurses in clinical practice.

Newcomers need to seek three types of information content related to task performance: technical information for acquiring job-related skills, referent information for understanding role demands and expectations, and normative information for understanding expected behaviors and attitudes [18]. For new graduate nurses, obtaining these three types of information content is essential for effective practice and for becoming integrated members of their wards. However, the support provided by the study participants remained at a fundamental level across the three types of information. It did not directly help new graduate nurses acquire job-related skills or understand their roles and expected behaviors. If nurse managers and senior nurses explicitly recognize these types of information needed by new graduate nurses, they may be able to facilitate information seeking more effectively, thereby promoting social integration into the ward [23].

Regarding information sources necessary for effective practice and professional development, three types of sources have been identified: textual, social, and physical [8]. New graduate nurses need to know where to find textual sources, such as manuals and information in electronic medical records, and how to access them. Accessing these sources is relatively easy because they have prior experience using them as nursing students. In line with this, a quantitative study conducted in Canada on the information-seeking behaviors of nursing students and new graduate nurses found that the most used sources were electronic resources, which fall under textual sources [32]. However, electronic medical record systems vary across hospitals; therefore, nurse managers in this study helped new graduate nurses locate necessary information within the records. In contrast, social sources that are related to becoming members of the team are more difficult to access. This is because the information derived from social sources is intangible and embedded in interpersonal relationships and everyday workplace interactions. Accessing such sources is difficult not only for new graduate nurses but also for nurse managers and senior nurses who try to support these nurses. Physical sources enable new graduate nurses to recognize their own bodies as sources of embodied knowledge. These sources are particularly crucial for nursing practice and are accessed primarily through observing other nurses in clinical settings. Therefore, senior nurses should recognize that their own practice constitutes an important social and physical information source for new graduate nurses’ information seeking.

According to an integrated model of employee information seeking [33], new graduate nurses must decide how to seek information by selecting appropriate tactics, sources, and timing within complex workplace contexts. Information-seeking tactics include direct questioning, observation, and consulting documents. For new graduate nurses, one of the most difficult decisions is determining the appropriate timing for asking questions in busy clinical situations. Accordingly, it is a useful strategy for senior nurses to adopt attitudes that facilitate information seeking by creating a supportive atmosphere and initiating conversations that make them approachable for consultation.

Passive and active information seeking are distinguished [10]. Although proactive information seeking is important for newcomers’ socialization, passively received information also contributes to task mastery and to role clarification. The study participants expected new graduate nurses to engage in proactive information seeking; however, they also provided information through identified information sources and explained what new graduate nurses needed to learn. Providing information to new graduate nurses helps them understand how to provide patient care and the behaviors expected of them as new graduate nurses. Because of the gap between how nursing students learn in school and how they are expected to practice [34,35,36], passive information seeking may be inevitable. Therefore, it is important for nurse managers and senior nurses to consider what kinds of information should be provided to new graduate nurses so as not to hinder their active learning.

The study participants, including both nurse managers and senior nurses, emphasized that new graduate nurses need to engage in information sharing with their peers, either online or in person. Based on a systematic review of peer support strategies for new graduate nurses, peer support enhances emotional resilience, job satisfaction, and retention by alleviating stress and feelings of isolation and by fostering shared learning and confidence building among new graduate nurses [37]. Sharing information with peers may therefore contribute not only to obtaining information but also to easing new graduate nurses’ transition experiences. However, when new graduate nurses rely excessively on peer information sharing, they may encounter gaps or limitations in the information they obtain due to their limited experience. This suggests that peer-based information exchange alone may not always provide sufficient or accurate guidance. Nurse managers and senior nurses should facilitate opportunities for peer information sharing while remaining attentive to the potential limitations of this strategy.

Among the identified expectations, the expectation that new graduate nurses demonstrate desirable attitudes toward seeking information, including respectful attitudes toward senior nurses and diligence as new graduate nurses, was not directly supported. A critical review of peer support for new graduate nurses reported that experienced nurses’ expectations of new graduate nurses emphasized the necessity of nursing skills, including clinical decision making, time management, patient assessment, and communication, as well as commitment to nursing and to their specific wards [38]. Among these expectations, only commitment to nursing was congruent with the present study’s finding regarding new graduate nurses’ diligence. In contrast to expectations reported in previous studies, the expectations expressed by participants in the present study were more focused on fundamental aspects of nursing practice. This difference may be explained by differences in research focus: whereas prior studies primarily examined expectations in terms of competence and readiness for practice, the present study focused on information seeking in everyday clinical practice. Consequently, expectations related to information seeking in immediate clinical practice tended to emphasize more basic attitudes. However, neither nurse managers nor senior nurses in the present study provided explicit guidance or education regarding these desirable attitudes. These attitudes appeared to be taken for granted and less amenable to direct support in busy clinical settings. This may explain why they were not directly reflected in the support described. Nevertheless, such attitudes are important for social acceptance, enabling new graduate nurses to feel liked and accepted by their peers, which corresponds to one dimension of workplace adjustment [39]. Therefore, basic nursing education should explicitly address and foster these attitudes before graduation, as they are likely to influence new graduate nurses’ adjustment to the clinical work environment [30].

Newcomer individual characteristics, such as personality traits, self-efficacy, proactive behavior, and emotions, shape how newcomers perceive their work environment, and these perceptions influence their information seeking [21]. As a result, support from nurse managers and senior nurses may be effective for some new graduate nurses but not for all. Direct support alone, therefore, is only one approach to facilitating new graduate nurses’ information seeking related to their integration into their wards. In this context, nurse managers’ indirect support is crucial. This includes creating a nurturing work environment, coordinating relationships within the ward, and supporting senior nurses’ educational roles. In addition, senior nurses’ efforts to foster attitudes that facilitate information seeking are also important for helping new graduate nurses understand when, from whom, and in what situations they can seek information.

This study has several limitations. Nurse managers and senior nurses’ support and expectations regarding new graduate nurses’ information seeking may differ depending on organizational members and organizational culture. Participants who agreed to be interviewed may have had a particular interest in supporting new graduate nurses’ information seeking; therefore, the level of support they described may be higher than that of other nurses. Furthermore, participants may have provided socially desirable responses regarding their support practices, as they were recommended by the directors of nursing, even though we assured them that their participation would remain confidential. This study employed a cross-sectional design with a single interview. Therefore, it provides a snapshot of participants’ experiences at a single point in time [40] and does not capture changes in support practices over time. In addition, qualitative descriptive designs focus on how phenomena occur rather than why, which may result in limited theoretical insight.

Despite these limitations, the findings of this study may be transferable to other hospital settings when considering how to support new graduate nurses’ information seeking. In particular, hospitals may consider implementing targeted interventions, such as workshops that promote desirable attitudes toward information seeking, including proactive communication, openness, and respect for senior nurses. In addition, providing regular opportunities for interaction between new graduate nurses and experienced nurses (e.g., regular meetings or reflective sessions) may facilitate access to information and enhance learning in clinical settings. Furthermore, nurse managers and senior nurses may benefit from training programs that emphasize strategies for proactively providing information and fostering supportive relationships with new graduate nurses.

Future research is needed to examine new graduate nurses’ own perceptions of information seeking and the support they receive. Qualitative longitudinal studies are needed to better understand new graduate nurses’ information-seeking process and the support practices of nurse managers and senior nurses.

## 5. Conclusions

This qualitative descriptive study explored nurse managers’ and senior nurses’ expectations and support for new graduate nurses’ information seeking, which is an important factor in organizational socialization. Nurse managers and senior nurses expected new graduate nurses to demonstrate appropriate attitudes toward seeking information, engage in proactive communication to express their needs or uncertainties, take initiative in seeking and obtaining information, and build relationships with senior nurses that enable information seeking.

Nurse managers’ indirect support involved creating a work environment in which all staff nurture new graduate nurses, mediating relationships between senior nurses and new graduate nurses, coordinating work assignments to promote interaction with various senior nurses, and supporting senior nurses in their educational roles. Their direct support included encouraging information sharing among peers, supporting new graduate nurses in expressing their own thoughts, providing information about colleagues, teaching information-seeking methods, and offering information tailored to new graduate nurses’ current level of practice.

Senior nurses’ support included adopting attitudes that facilitate information seeking, proactively providing necessary information, adapting teaching strategies to promote information seeking, and encouraging information sharing among peers. Overall, nurse managers and senior nurses need to foster a supportive work environment and recognize the information content, sources, tactics, and timing required for effective support.

## Figures and Tables

**Table 1:** Characteristics of the study participants: Nurse managers.

Participant	Sex	Age	Managerial Experience	Ward	Hospital
1	Female	49	19	Convalescent rehabilitation	A
2	Female	38	6	Mixed	A
3	Female	50	2	Community-based integrated care	B
4	Female	45	5	Mixed	A
5	Female	55	3	Internal medicine and pediatrics	B
6	Female	53	4	community-based integrated care	B
7	Female	47	3	Comprehensive surgical center	B
8	Female	47	3	Mixed	C
9	Female	54	16	Orthopedics and otolaryngology	D
10	Female	58	12	Gastroenterology	D
11	Female	53	9	Mixed	E
12	Female	51	7	Mixed	C

Hospitals A and B: Acute care hospitals with post-acute and community care wards; Hospitals C, D, and E: Acute care hospitals.

**Table 2:** Characteristics of the study participants: Senior nurses.

Participant	Sex	Age	Nursing Experience	Ward	Role	Hospital
1	Female	38	18	Mixed	Charge nurse	A
2	Female	29	6	Convalescent rehabilitation	Preceptor	A
3	Female	44	21	Convalescent rehabilitation	Charge Nurse *	A
4	Female	31	9	Mixed	Charge Nurse *	A
5	Female	28	7	Comprehensive surgical center	Preceptor	B
6	Female	31	6	Community-based integrated care	Ward-based Nurse Educator	B
7	Female	31	10	Community-based integrated care	Preceptor	B
8	Male	46	25	High care unit	Charge Nurse *	B
9	Female	46	21	High care unit	Charge Nurse *	B
10	Male	25	3	General internal medicine	Preceptor	B
11	Male	40	6	Gastroenterology	Ward-based Nurse Educator	D
12	Female	48	27	Female-Only	Charge nurse	D
13	Female	41	15	OB-GYN and Pediatric	Charge nurse	D
14	Female	49	25	Female-Only	Charge nurse	D

Hospitals A and B: Acute care hospitals with post-acute and community care wards; Hospital D: Acute care hospitals. * Additional responsibilities as hospital education coordinators.

**Table 3:** Expectations for New Graduate Nurses’ Information Seeking.

Categories	Subcategories
Demonstrating desirable attitudes toward seeking information	Showing willingness to learn
	Receiving guidance with a respectful attitude
	Demonstrating diligence as a new graduate nurse
Engaging in proactive communication to express one’s needs or uncertainties	Expressing one’s uncertainties or difficulties verbally
	Asking questions proactively when unsure
	Voicing one’s own thoughts even when unsure
	Communicating needed information to senior nurses
Taking initiative to seek and obtain information	Actively sharing information with peers
	Looking up unknown information independently
	Obtaining necessary information while collecting it with senior nurses
	Identifying information-gathering methods suited to oneself
Building relationships with senior nurses to enable information seeking	Making attempts to initiate conversations with senior nurses
	Sharing personal information during casual conversations

**Table 4:** Nurse Managers’ Direct Support for New Graduate Nurses’ Information Seeking.

Categories	Subcategories
Encouraging information sharing among peers	Encouraging information sharing among peers
Supporting new graduate nurses to express their own thoughts	Supporting new graduate nurses to express their own thoughts
Providing information about colleagues	Explaining senior nurses’ roles and responsibilities
	Providing information about physicians’ characteristics
Teaching information-seeking methods	Teaching who to consult when unsure
	Teaching how to search for information in the electronic medical records
	Teaching which reference materials to consult
Providing information tailored to new graduate nurses’ current practice level	Providing information tailored to new graduate nurses’ current practice level

**Table 5:** Senior Nurses’ Support for New Graduate Nurses’ Information Seeking.

Categories	Subcategories
Adopting attitudes that facilitate information seeking	Creating a comfortable atmosphere for information seeking
	Initiating conversations to make oneself approachable for consultation
Providing necessary information proactively	Taking the initiative to share information
	Explaining what new graduate nurses need to learn
	Searching for information together
	Teaching where information sources are located
Adapting teaching strategies to promote information seeking	Considering the characteristics of new graduate nurses when providing guidance
	Verbalizing one’s own thinking while providing guidance
	Checking understanding by asking questions about what new graduate nurses have learned
Encouraging information sharing among peers	Encouraging information sharing among peers

## Data Availability

The original contributions presented in this study are included in the article. Further information may be obtained from the corresponding author.

## Supplementary Materials

The following supporting information can be downloaded at: https://www.mdpi.com/article/10.3390/nursrep16050153/s1, Table S1: COREQ (COnsolidated criteria for REorting Qualitative research) checklist.

## Public Involvement Statement

No public involvement in any aspect of this research.

## Guidelines and Standards Statement

This manuscript was drafted against the Table S1 COREQ (Consolidated criteria for REporting Qualitative research) checklist for qualitative research.
