# Supplementary material for: Supporting New Graduate Nurses’ Information Seeking: Perspectives of Nurse Managers and Senior Nurses in Japanese Hospitals
Source: Nurs Rep. 2026 Apr 30;16(5):153. doi: 10.3390/nursrep16050153 (PMC13209906; doi:10.3390/nursrep16050153)
Supplement: Supplementary file 1 [file nursrep-16-00153-s001.zip › nursrep-4189906-supplementary.pdf]

Table S1. COREQ (COnsolidated criteria for REorting Qualitative research) checklist

Item	Item No.	Guide Questions / Description	Reported on Page No.
**Domain 1: Research Team and Reflexivity**			
Personal Characteristics			
Interviewer/facilitator	1	Which author/s conducted the interview or focus group?	4
Credentials	2	What were the researcher's credentials? E.g. PhD, MD	4
Occupation	3	What was their occupation at the time of the study?	3
Gender	4	Was the researcher male or female?	4
Experience and training	5	What experience or training did the researcher have?	5
Relationship with participants			
Relationship established	6	Was a relationship established prior to study commencement?	4
Participant knowledge of the interviewer	7	What did the participants know about the researcher? e.g. personal goals, reasons for doing the research	4
Interviewer characteristics	8	What characteristics were reported about the inter viewer/facilitator? e.g. Bias, assumptions, reasons and interests in the research topic	4
**Domain 2: Study Design**			
Theoretical framework			
Methodological orientation and theory	9	What methodological orientation was stated to underpin the study? e.g. grounded theory, discourse analysis, ethnography, phenomenology, content analysis	3
Participant selection			
Sampling	10	How were participants selected? e.g. purposive, convenience, consecutive, snowball	3
Method of approach	11	How were participants approached? e.g. face-to-face, telephone, mail, email	3
Sample size	12	How many participants were in the study?	5
Non-participation	13	How many people refused to participate or dropped out? Reasons?	3
Setting			
Setting of data collection	14	Where was the data collected? e.g. home, clinic, workplace	4
Presence of non-participants	15	Was anyone else present besides the participants and researchers?	4
Description of sample	16	What are the important characteristics of the sample? e.g. demographic data, date	5-7

Data collection			
Interview guide	17	Were questions, prompts, guides provided by the authors? Was it pilot tested?	4
Repeat interviews	18	Were repeat interviews carried out? If yes, how many?	N/A
Audio/visual recording	19	Did the research use audio or visual recording to collect the data?	4
Field notes	20	Were field notes made during and/or after the interview or focus group?	4
Duration	21	What was the duration of the inter views or focus group?	6
Data saturation	22	Was data saturation discussed?	5
Transcripts returned	23	Were transcripts returned to participants for comment and/or correction?	N/A
**Domain 3: Analysis and Findings**			
Data analysis			
Number of data coders	24	How many data coders coded the data?	4
Description of the coding tree	25	Did authors provide a description of the coding tree?	7-13
Derivation of themes	26	Were themes identified in advance or derived from the data?	4
Software	27	What software, if applicable, was used to manage the data?	4
Participant checking	28	Did participants provide feedback on the findings?	5
Reporting			
Quotations presented	29	Were participant quotations presented to illustrate the themes/findings?	7-13
Data and findings consistent	30	Was each quotation identified? e.g. participant number	7-13
Clarity of major themes	31	Was there consistency between the data presented and the findings?	7-13
Clarity of minor themes	32	Is there a description of diverse cases or minor themes?	N/A
